# Multi-Omics Investigation of Innate Navitoclax Resistance in Triple-Negative Breast Cancer Cells

**DOI:** 10.3390/cancers12092551

**Published:** 2020-09-08

**Authors:** Michal Marczyk, Gauri A. Patwardhan, Jun Zhao, Rihao Qu, Xiaotong Li, Vikram B. Wali, Abhishek K. Gupta, Manoj M. Pillai, Yuval Kluger, Qin Yan, Christos Hatzis, Lajos Pusztai, Vignesh Gunasekharan

**Affiliations:** 1 Yale Cancer Center, Yale School of Medicine, New Haven, CT 06511, USA; michal.marczyk@yale.edu (M.M.); gpatward@its.jnj.com (G.A.P.); xiaotong.li@yale.edu (X.L.); vwali1@its.jnj.com (V.B.W.); abhishek.gupta@yale.edu (A.K.G.); manoj.pillai@yale.edu (M.M.P.); christos.hatzis@yale.edu (C.H.); vignesh.gunasekharan@yale.edu (V.G.); 2 Department of Data Science and Engineering, Silesian University of Technology, 44-100 Gliwice, Poland; 3 Computational Biology & Bioinformatics Program, Yale University, New Haven, CT 06511, USA; jun.zhao@yale.edu (J.Z.); rihao.qu@yale.edu (R.Q.); yuval.kluger@yale.edu (Y.K.); 4 Department of Pathology, Yale School of Medicine, New Haven, CT 06511, USA; qin.yan@yale.edu

**Keywords:** navitoclax, drug resistance, cancer therapy, signature, multi-omics, triple-negative breast cancer

## Abstract

**Simple Summary:**

Triple negative breast cancer is a disease with limited treatment options and the poorest outcome across all breast cancer subtypes, thus the need for new effective therapies is high. We recently found that navitoclax displays synergistic anti-proliferative and apoptotic activities with other drugs in treatment of triple negative breast cancer cells, but the resistance to treatment is still a limiting factor. Therefore, we investigated the effects of navitoclax treatment on the transcriptome, genome and epigenome in vitro to better understand the process of developing resistance. We discovered and validated a list of multiple, previously unknown markers of drug resistance that can help in patient selection in future clinical trials involving navitoclax.

**Abstract:**

Cancer cells employ various defense mechanisms against drug-induced cell death. Investigating multi-omics landscapes of cancer cells before and after treatment can reveal resistance mechanisms and inform new therapeutic strategies. We assessed the effects of navitoclax, a BCL2 family inhibitor, on the transcriptome, methylome, chromatin structure, and copy number variations of MDA-MB-231 triple-negative breast cancer (TNBC) cells. Cells were sampled before treatment, at 72 h of exposure, and after 10-day drug-free recovery from treatment. We observed transient alterations in the expression of stress response genes that were accompanied by corresponding changes in chromatin accessibility. Most of these changes returned to baseline after the recovery period. We also detected lasting alterations in methylation states and genome structure that suggest permanent changes in cell population composition. Using single-cell analyses, we identified 2350 genes significantly upregulated in navitoclax-resistant cells and derived an 18-gene navitoclax resistance signature. We assessed the navitoclax-response-predictive function of this signature in four additional TNBC cell lines in vitro and in silico in 619 cell lines treated with 251 different drugs. We observed a drug-specific predictive value in both experiments, suggesting that this signature could help guiding clinical biomarker studies involving navitoclax.

## 1. Introduction

Cytotoxic insults induce a complex cellular stress response that enables cancer cells to survive [1]. High throughput molecular analytical techniques allow us to simultaneously and comprehensively study multiple aspects of the dynamic stress response, both at the cell population and single-cell levels. Previous studies have documented extensive gene expression changes in cancer cells after in vitro exposure to various cancer drugs [2,3,4]. Tissue environmental stress induced by treatment also leads to changes in chromatin accessibility and DNA methylation [5,6]. Furthermore, clonal selection due to treatment pressure has been widely observed with either enrichment or loss of various genomic features of surviving cells in both experimental models and clinical samples [7]. By simultaneously analyzing all the different genetic and epigenetic factors, we can better understand the process of drug resistance development and design new therapeutic strategies to overcome resistance.

The balance between pro-survival (Bcl-2, Bcl-X_L_, Bcl-w, Mcl-1, and A1) and pro-apoptotic (Bax, Bak, Bim, Bid, Puma, Bad, Noxa, Bik, Bmf, and Hrk) proteins largely determines whether a cell lives or dies after various types of cytotoxic insults [8]. Overexpression of pro-survival proteins in cancer generally correlates with poor prognosis and resistance to chemotherapy. Navitoclax (ABT-263) disrupts protein–protein interactions between the pro-survival proteins, including Bcl-2, Bcl-X_L_ (coded by the *BCL2L1* gene) and Bcl-w (coded by the *BCL2L2* gene) molecules and the pro-apoptotic family of proteins, leading to unopposed pro-apoptotic signaling [9,10]. In vivo testing of navitoclax in human trials showed a decrease in platelet counts that resulted from Bcl-X_L_ inhibition, however, thrombocytopenia can be controlled by appropriate dosing [11]. As a single agent, navitoclax showed limited activity against advanced and recurrent small-cell lung cancer [12], but it showed synergistic activity in combination with gemcitabine in solid tumors [13], with brentuximab in Hodgkin’s lymphoma [14], with enzalutamide in castration-resistant prostate cancer [15], and with T-DM1 in HER2-positive breast cancer [16]. Currently, navitoclax is being tested in multiple ongoing clinical trials on various cancer types (https://clinicaltrials.gov/).

Intrinsic genomic and molecular differences between different breast cancer subtypes explain their distinct clinical course and general differences in their drug sensitivities [17,18,19]. The breast cancer subtype with the least therapeutic options and therefore the poorest outcome is triple-negative breast cancer (TNBC) [20]. Immunotherapy, antibody drug conjugates, and PARP inhibitors recently emerged as new treatment options for subsets of TNBC, but new effective therapies are still needed. We recently demonstrated that crizotinib and navitoclax displayed synergistic anti-proliferative and apoptotic activities in TNBC cells in vitro [21]. In the current study, we focus on investigating the effects of navitoclax treatment on the transcriptome (single-cell and bulk RNA sequencing (RNAseq)), methylome (bisulphite sequencing), chromatin structure (assay for transposase-accessible chromatin sequencing (ATACseq)), and DNA copy number alterations (shallow whole genome sequencing) of MDA-MB-231 TNBC cells. This cell line model was selected based on our previous work investigating the combination of navitoclax with multiple other drugs in TNBC cell lines [21]. Although we focus on a single cell line, we employ a very broad and comprehensive longitudinal strategy to examine the contribution and dynamics of multiple biological processes to the treatment response. We studied the multi-omics response of cells at the population level at baseline before treatment, at the end of 72 h of navitoclax exposure, and after 10-day drug-free recovery from treatment. This treatment schedule gave the highest and quickest cancer cell re-growth after end of drug treatment among the 696 treatment schedules that were tested in our preliminary study [22]. Since we wanted to study the emergence of resistant mechanisms, we used relative IC_90_ dose of single agent navitoclax (10 µM) that allows survival of approximately 50% of the cell population. Our goal was to identify the molecular changes that characterize the cells that survived treatment and to examine whether these changes represent a transient stress response or prolonged genomic, epigenetic, or transcriptional alterations that become fixed in the resistant cells. We also performed single-cell RNA sequencing to study heterogeneity in transcriptional response across cells and used this data to generate an 18-transcript navitoclax resistance signature, which was further tested for association with navitoclax response in four triple-negative breast cancer cell lines in vitro and in over 600 cancer cell lines of various tissue origins in silico.

## 2. Results

**Figure 1:**
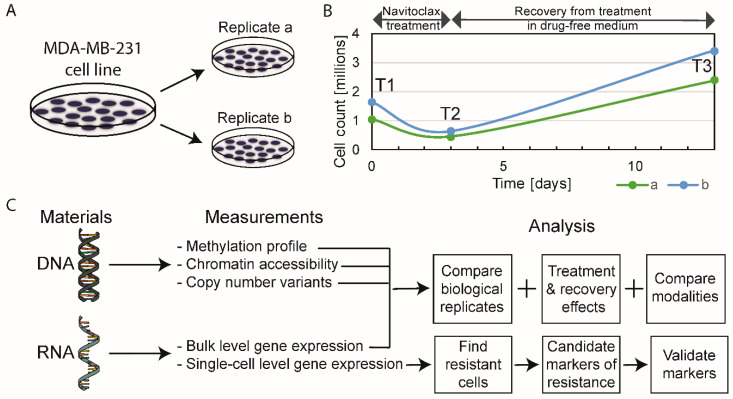
Schematic of experimental design. (**A**) Biological replicates, experiments “a” and “b”, were performed simultaneously. (**B**) Absolute cell counts before treatment (T1, baseline), at the end of 72-h of exposure to navitoclax (T2, on-treatment sample) and after 10-day drug-free recovery from treatment (T3, post-treatment sample). (**C**) The type of omics analysis at each time point.

MDA-MB-231 cells were grown in two identical parallel experiments (i.e., biological replicates), termed replicates “a” and “b”. Cells were harvested for omics analyses at three time points—T1: before treatment (or baseline), T2: after 3 days of 10-µM navitoclax exposure (on-treatment), and T3: after 10 days of recovery in drug-free medium post-3-day treatment (post-treatment) (Figure 1A,B). Five different types of molecular analyses were performed at all time points (Figure 1C). For each omics modality, we assessed on-treatment effects (T2 versus T1), post-treatment recovery effects (T3 versus T2), and persistent resistance effects (T3 versus T1).

### 2.1. The Effect of Navitoclax Treatment on MDA-MB-231 Cell Growth and Concordance of Results in Replicate Measurements

Approximately 40% of the initial cell population (absolute cell counts, not normalized to control) survived the 72-h treatment with navitoclax, but doubled compared to baseline after the 10-day drug-free post-treatment period (Figure 1B). Correlation coefficients between biological replicates at different time points were high for all omics modalities, ranging between 0.97–1.00 for bulk RNAseq, 0.81–0.86 for single-cell RNAseq, 0.85–0.9 for DNA methylation, 0.74–0.95 for ATACseq, and 0.97–0.99 for copy number variants (CNVs) (Figure S1A). On average, the correlation of gene expression levels was higher for bulk RNA compared to single cell data (average Spearman correlation coefficient (r): 0.99 vs. 0.83). Overall, these results indicate good reproducibility and consistency of omics measurements. We observed higher than average deviations in replicate ATACseq results at baseline (T1 time point). The differences observed in biological replicates likely represent a combination of genuine biological differences between parallel cultures and technical noise in the measurements. The correlation between biological replicates at each time point was greater than the average correlation between different time points, which indicates that biological information can be gained from these results (Figure S1B).

### 2.2. Multi-Omics Effects of 72 h of Navitoclax Exposure

**Figure 2:**
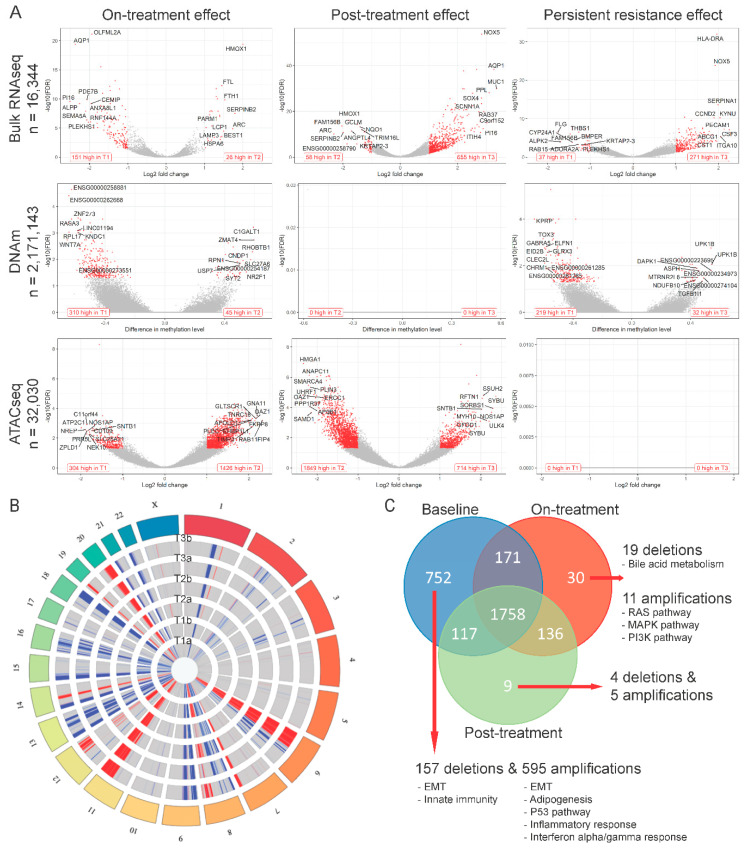
Treatment effects at individual gene level per modality. (**A**) Volcano plots showing genes with differential expression level (top), methylation level (middle), or peak abundance (bottom) for on-treatment (T2 versus T1), post-treatment recovery (T3 versus T2), and persistent resistance effects (T3 versus T1). Red dots represent significantly affected genes (see Methods). In lower corners of each plot the number of up- and downregulated genes is presented using red text labels. (**B**) Circos plot showing genome regions with copy number deletions (blue) and amplifications (red) in each analyzed sample. (**C**) Venn diagram comparing genes with copy number variants (CNVs) found in each treatment phase. Red arrows point to results of over-representation analysis of treatment-exclusive CNVs.

**Figure 3:**
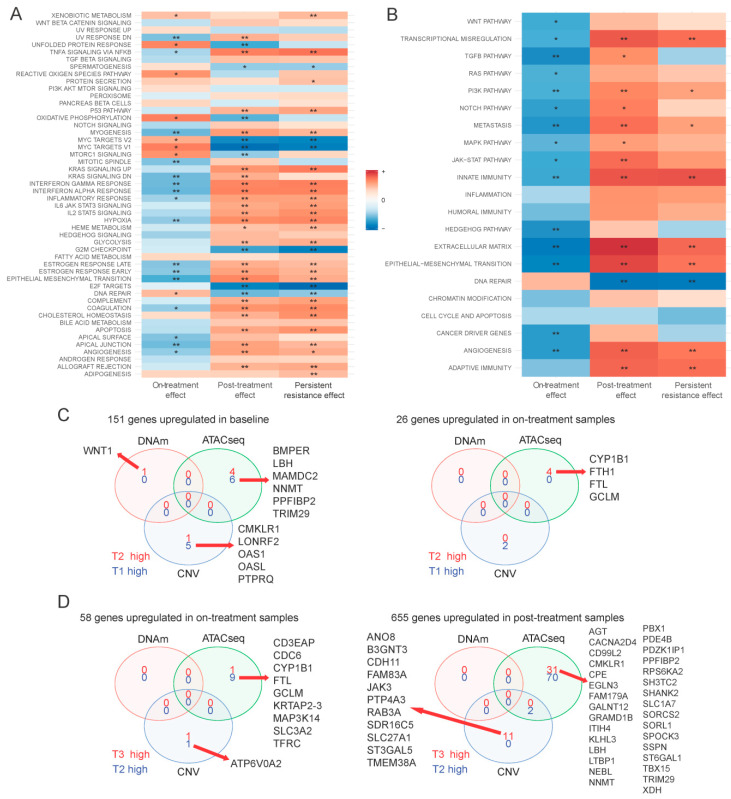
Functional analysis and multi-omics comparison. Gene set enrichment analysis of bulk RNAseq data on MSigDB hallmark genes (**A**) and Nanostring cancer hallmark pathways (**B**). Color indicates directionality, e.g., for on-treatment effect + means overexpression of gene set after 72-h treatment (in T2 vs. T1). Stars indicate significant results (* adjusted *p* < 0.01, ** adjusted *p* < 0.001). (**C**) Venn diagram of genes overexpressed at baseline (left) and after 72-h treatment (right) samples in on-treatment effects comparison and genes affected by methylation, chromatin structure changes, and CNV. (**D**) Venn diagram of genes overexpressed after 72-h treatment (left) and after 10-day drug-free period (right) samples in post-treatment effect comparison and genes affected by methylation, chromatin structure change, and CNV. For DNA methylation (DNAm), “high” means hyper-methylation. For assay for transposase-accessible chromatin sequencing (ATACseq), “high” means more opened chromatin structure. For CNV, “high” means amplification when there was no CNV in other groups, or no CNV when there was deletion in other groups.

Using bulk RNAseq data, we compared differentially expressed genes between the baseline populations (T1a, T1b) and the surviving cells after 72 h of drug treatment (T2a, T2b). Twenty-six genes showed significantly higher expression and 151 showed lower expression in the on-treatment (T2) samples (Figure 2A and Table S1). Genes upregulated at 72 h were enriched in MYC targets, oxidative phosphorylation and reactive oxygen species (ROS) pathways, whereas the downregulated genes were enriched in hypoxia, epithelial-mesenchymal transition (EMT), interferon alpha response and myogenesis pathways (Figure 3A,B). This suggests an increase in mitochondrial biogenesis and increased metabolic activity in surviving cells. We also identified 310 DNA regions (CpG sites) that were hypo-methylated and 45 that showed higher methylation in T2 samples compared to baseline. Only 1 of the 151 genes overexpressed at baseline showed significant hypomethylation at baseline, but 55% showed some degree of statistically non-significant hypo-methylation. None of the 26 genes overexpressed in T2 samples showed significant hypo-methylation at the corresponding time point, but 46% showed statistically non-significant hypo-methylation (Figure 3C). These findings suggest that methylation changes only explain a minority of navitoclax-induced gene expression changes at 72 h. However, on average, the difference in the methylation levels of the promoter regions of the genes differentially expressed between T1 and T2 was negatively correlated with gene expression levels, which confirms a small, but consistent influence of hypo-methylation on higher gene expression level. The correlation was even stronger (Δ= −0.22 vs. Δ= −1.02) in the group of genes that showed statistically significant differences in expression between the two time points (Figure S2A). When we examined chromatin accessibility using ATACseq, 304 sites showed greater chromatin openness in baseline samples, including transcriptional regulatory regions of six of the 151 genes with higher expression at baseline (65% showed numerically higher but not statistically significant chromatin openness). We observed significantly higher chromatin openness, affecting 1426 sites in T2 samples compared to baseline. This was surprising, since only 26 genes showed higher expression at the T2 time point and only four of these had increased open chromatin structure in T2 compared to baseline (Figure 3C). This could also indicate a delayed effect of the chromatin changes, since a very large number of genes started to show overexpression during the recovery period after 72 h of drug exposure (see next section of results). Furthermore, on average, the difference in the chromatin accessibility of gene coding regions was positively correlated with the change in gene expression levels. The correlation was stronger (Δ = 0.07 vs. Δ = 0.5) in a group of genes with significant change in expression (Figure S2B). These data indicate that while navitoclax treatment induced large scale chromatin opening, this did not translate into a large increase in mRNA expression in the affected genes. At the copy number level (Figure 2B), we found 78 genes amplified in T2 samples compared to baseline, affecting none of the 26 genes overexpressed in T2, and 88 genes were deleted in T2 compared to baseline, affecting five of the 151 lower expressed genes (Figure 2C). One hundred and seventy two genes showed deletions and 697 genes showed amplification at baseline, but not in the T2 samples, indicating that cells with these genomic alterations were eliminated by the treatment.

### 2.3. Multi-Omics Changes after 10 Days of Drug-Free Recovery

When we compared mRNA expression between surviving cells at 72 h (on-treatment T2 samples) and cells after the 10-day recovery period (post-treatment T3 samples), there were 655 overexpressed genes and 58 with lowered expression in the post-treatment population (Figure 2A and Table S1). Overexpressed genes were enriched in glycolysis, hypoxia, apoptosis, TNF-α signaling via NF-κβ, interferon alpha/gamma response, and EMT transition pathways, whereas the downregulated genes were enriched in oxidative phosphorylation, ROS signaling, MYC targets, E2F targets and G2M checkpoint pathways (Figure 3A,B). These findings suggest that during recovery, most of the cells reverse towards a pretreatment basal state and only some cell subpopulations have retained treatment-associated gene expression changes. We observed no methylation changes post-treatment during the recovery period, but continued to see large scale chromatin structure changes, with substantially more regions having open chromatin at 72 h (*n* = 1849) compared to the end of the 10-day post-treatment recovery period (*n* = 714) (Figure 2A and Table S1). Despite the absence of drug-mediated selection pressure on the cells, we also detected additional CNV changes (Figure 2C) with 106 genes amplified and 20 genes deleted post-treatment (in T3, but not in T2), indicating delayed elimination of irreversibly damaged cells. Again, DNA methylation, copy number, and chromatin changes only explained a minority of the observed gene expression differences (Figure 3D).

We also compared the transcriptional and genomic changes between baseline (T1) and post-treatment recovery samples (T3) (Figure 2A and Table S1) to assess how the re-expanded surviving cell population differed from the starting population. The overall gene expression correlations between T1 and T3 samples were very high (r = 0.94 and 0.99 for the two replicate samples), indicating highly similar transcriptional states before treatment and post-recovery. Of the 26 genes upregulated at 72 h of drug exposure, 24 returned to baseline level by the end of the recovery period, and 146 of the 151 downregulated genes also returned to baseline level. However, there were 271 genes with higher and 37 with lower expression in T3 samples compared to baseline. These genes represented essentially the same biological pathways as those altered in the on-treatment (T2) samples (Figure 3A,B). We also observed DNA methylation differences, including 219 sites showing hypo- and 32 showing hyper-methylation levels in T3 samples compared to T1. These methylation changes might represent long-lasting epigenetic changes after cellular stress. All the large-scale chromatin structure changes that were observed after 72 h of drug exposure were normalized by the end of the drug-free recovery phase. There were no significant differences in chromatin openness between T1 and T3 samples (Figure 2A and Table S1). DNA copy number changes (Figure 2B) selected for, or acquired, during treatment also persisted after the recovery phase, indicating permanent elimination of certain cells. Overall, 60% of genes with CNVs were shared across all three time points (Figure 2C), but we found 71 genes amplified post-treatment, but not at baseline, and 74 genes with deletion in post-treatment samples, but not at baseline. Interestingly, 752 genes with CNVs were only found in baseline samples, suggesting that cells carrying these CNVs were highly sensitive to navitoclax. Of the 752 genes, 157 were deleted in baseline samples and these genes were enriched in EMT and innate immunity pathways, and 595 genes were amplified in baseline samples and were enriched in EMT, adipogenesis, P53, inflammatory, and interferon alpha/gamma response pathways.

### 2.4. Single-Cell Assessment of Changes in Cell-Cycle States and Expression Changes of Known Navitoclax Response-Related Genes

**Figure 4:**
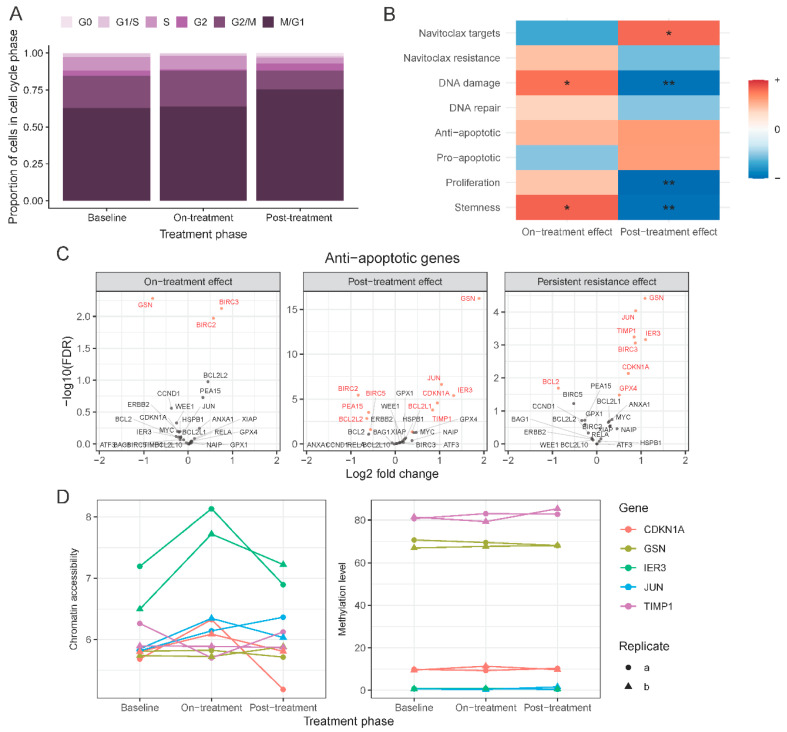
Gene set level changes between treatment phases. (**A**) Distribution of cells across cell-cycle phases in each treatment phase based on single-cell expression data. (**B**) Changes in the expression level of genes grouped in 8 biologically relevant signatures found by gene set enrichment analysis. * indicates enrichment at 0.01 and ** at 0.001 significance level. (**C**) Volcano plots of anti-apoptotic genes in 3 comparisons: on-treatment vs. baseline (left), post-treatment vs. on-treatment (middle), post-treatment vs. baseline (right). Red indicates significant change in expression (FDR < 0.05). (**D**) Chromatin accessibility level (left) and DNA methylation level (right) of selected anti-apoptotic genes in each treatment phase.

Single-cell RNAseq allowed us to assess the effects of treatment at the individual cell level, and more specifically to assess treatment-related changes in the proliferation state of cells compared to baseline (Figure 4A). We used a set of cell cycle associated genes (*n* = 540) to assign cells into G0, G1/S, S, G2, G2/M, and M/G1 cell cycle phases. After 72 h of navitoclax treatment, there was a small but significant increase of cells in the G2/M phase compared to baseline (24% on-treatment vs. 22% at baseline, *p* = 0.02). At the end of the 10-day post-treatment recovery, there were more cells in G0 (1.7% post-treatment (T3) vs. 0.4% on-treatment (T2) and 0.3% at baseline (T1; *p* = 3 × 10^−5^, *p* = 5 × 10^−6^, respectively) and M/G1 state (75% post-treatment vs. 64% on-treatment and 63% at baseline; *p* = 4 × 10^−17^, *p* = 2 × 10^−19^, respectively).

Next, we examined expression changes in known navitoclax response-related genes in T2 and T3 samples using the bulk RNAseq data (Figure 4B). These genes were curated from the published literature and included genes that are direct or indirect targets of navitoclax (*n* = 81), and genes associated with navitoclax resistance (*n* = 61), involved in DNA damage (*n* = 108) and DNA repair (*n* = 31), anti-apoptotic genes (*n* = 26), pro-apoptotic genes (*n* = 54), proliferation markers (*n* = 32), and cancer stem cell associated genes (*n* = 109) (Table S2). On average, the expression of navitoclax targets decreased on-treatment (T2) compared to baseline (false discovery rate (FDR) = 0.08) and increased in post-treatment (T3) samples (FDR = 0.003). An opposite but non-significant trend was observed for previously published markers of resistance to navitoclax. Cells that survived treatment, T2 samples, had elevated expression of DNA damage genes (FDR = 0.005). There were no significant changes in sets of DNA repair, anti- or pro-apoptotic genes. However, seven anti-apoptotic genes, BIRC3, CDKN1A, GPX4, GSN, IER3, JUN, and TIMP1, had significantly elevated expression in T3 compared to baseline, whereas BCL2 was reduced in T3 samples (Figure 4C). This shift in expression was not driven by statistically significant alterations in DNA methylation, chromatin accessibility, or copy number changes at these alleles (Figure 4D). Lastly, we observed increased levels of stemness markers in the on-treatment cells (T2) (FDR < 0.01), which returned to baseline levels after the 10-day post-treatment recovery period (T3) (FDR < 0.001).

### 2.5. Development of a Novel Navitoclax Resistance Gene Expression Signature from Single-Cell Analysis

**Figure 5:**
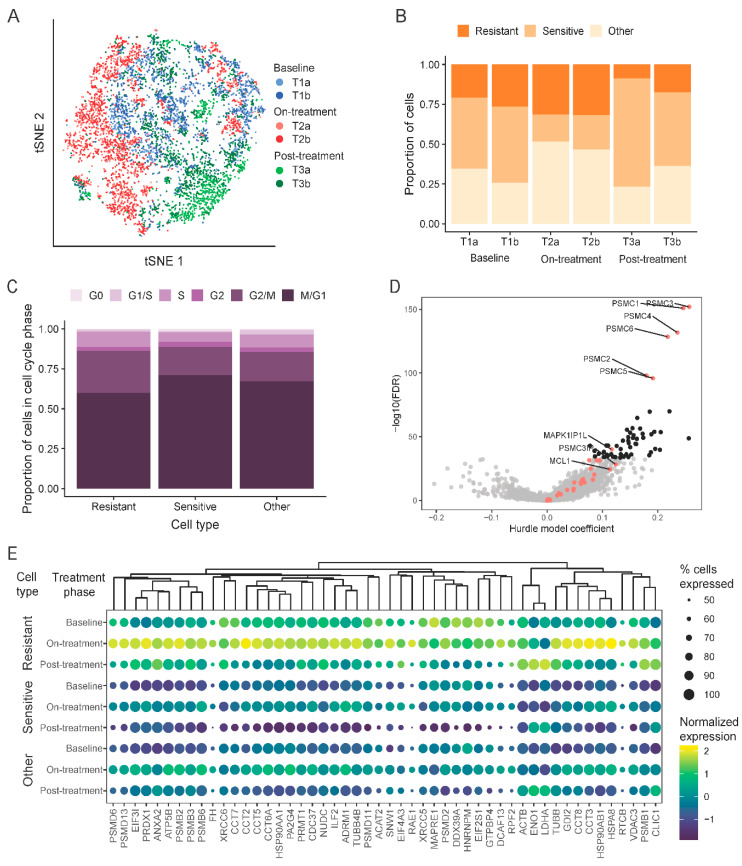
Single-cell RNAseq used to find drug resistant cells. (**A**) t-distributed stochastic neighbor embedding (t-SNE) plot of aggregated data colored by sample (T1a, T1b: baseline; T2a, T2b: on-treatment; T3a, T3b: post-treatment). (**B**) Proportion of cells characterized as sensitive or resistant to navitoclax within each sample. (**C**) Cell-cycle phase distribution within cell subpopulations stratified by resistance or sensitivity to navitoclax. (**D**) Volcano plot showing comparison of drug resistant cells to all other cells. Red dots are navitoclax-resistant genes in the gene set used for classification. Black dots are new markers of resistance shown in panel (**E**). (**E**) Expression of top 50 new markers of resistance to navitoclax treatment in different types of cells and samples. Dendrogram was created by hierarchical clustering with Euclidean distance. The size of the dots represents the proportion of expressed cells within a group. Gene expression was normalized by computing counts per million and log transformation.

When all the transcripts were considered across all six samples (three treatment points × two biological replicates) at the single-cell level, no distinct cell clusters that correspond to a single treatment point emerged (Figure 5A). However, there were some sets of cells in the on-treatment phase (T2) and post-treatment phase (T3) that grouped close to each other and separate from others, whereas baseline cells were more spread out. Overall, these results indicate that the majority of transcripts remained stable in all three sets of samples, which is consistent with the bulk sequencing data. It also indicates that the relatively small-scale gene expression changes induced by navitoclax in T2 and T3 samples were not consistently picked up at the single-cell level. Transcript diversity in cells across all time points showed the highest consistency (i.e., similarity) in gene expression between cells in on-treatment T2 samples (Jensen–Shannon divergence equals 0.12). We hypothesize that cells at T2 acquire a more similar transcriptomic state because of limited proliferative activity, whereas cells grown in drug-free media (T1 and T3) are more dynamic and show more varied transcriptomic profiles.

To identify transcriptomic cell transitions at the single-cell level between treatment points, the partition-based graph abstraction (PAGA) algorithm was applied. Fourteen cell clusters were identified (Figure S3A,B) that each contained a variable number of cells from all three treatment time points (Figure S3C). Clusters 1, 2, 3, 5, 10, and 11 consisted mostly of on-treatment surviving T2 cells; clusters 4, 7, 9, 12, and 13 mostly of baseline cells (T1); and clusters 0, 6, and 8 mostly of post-treatment cells (T3). These results indicate that sensitive and resistant cells co-exist at each treatment time point. We also observed a distinct cluster (cluster 7), which included predominately baseline cells (T1) and showed a distinct transcriptomic profile, unrelated to any other clusters.

To track dynamic changes in the proportions of presumed navitoclax-sensitive and resistant cells through the three treatment time points, we assigned individual cells into one of three categories: resistant, sensitive, or other (Figure 5B), using the expression levels of the literature-derived navitoclax resistance and navitoclax target genes (algorithm described in Methods section). Navitoclax resistance genes were those reported to be overexpressed in cells not responding to treatment with different anti-apoptotic protein inhibitors. Using this method, at baseline (T1), 46% of cells were classified as treatment-sensitive and about 23% as resistant. We observed a significant decrease in sensitive cells (19%; *p* ≤ 0.001) and enrichment of resistant cells (32%) in the on-treatment T2 samples. The post-treatment, post-recovery samples (T3) contained a low proportion of resistant cells (14%) and a high proportion of treatment-sensitive cells (56%), similar to baseline (T1). This is consistent with the bulk RNAseq results that also showed overall gene expression patterns returning to baseline after the recovery period. These results suggest that drug resistance is not permanent after a brief exposure to navitoclax, and a second cycle of treatment might be as effective against the surviving recovered cells as the first cycle was against the starting cell population. On the PAGA graph, the main resistant subpopulation is captured in cluster 11 (Figure S3E), which includes cells from all three time points (Figure S3C), whereas the most sensitive subpopulation was in cluster 13, which encompasses cells almost exclusively from the baseline and post-treatment post-recovery samples (Figure S3C,D). Cluster 11 is directly connected to a group of clusters consisting mostly of cells after treatment T2, but it also includes baseline cells (T1), suggesting that some cells at baseline possess a transcriptional state that is similar to cells that are were exposed to navitoclax and survived the insult.

By analyzing the cell cycle states of the resistant, sensitive, and remaining cells (Figure 5C), we observed that a significant number of resistant cells were in a proliferative state. Assuming that all resistant cells, irrespective of whether they are found in T1, T2 or T3 samples, share gene expression features that mediate resistance, we compared the gene expression levels between the cells classified as resistant to all other cells (Figure 5D and Table S3). We found 2350 genes with significant overexpression in resistant cells and 572 with significantly lower expression. Among genes enriched in resistant cells, we found 27 known markers and numerous other genes previously not linked to navitoclax response (black dots in Figure 5D represent top 50 markers). Figure 5E shows the expression of these new resistance markers in each sample. The genes overexpressed in resistant subpopulations were enriched for MYC and E2F targets and were involved in angiogenesis and the JAK-STAT pathway, whereas the genes with a lower expression were enriched in the Hedgehog signaling pathway (Figure S4A,B).

### 2.6. Validation of the Navitoclax Resistance Signature In Vitro

**Figure 6:**
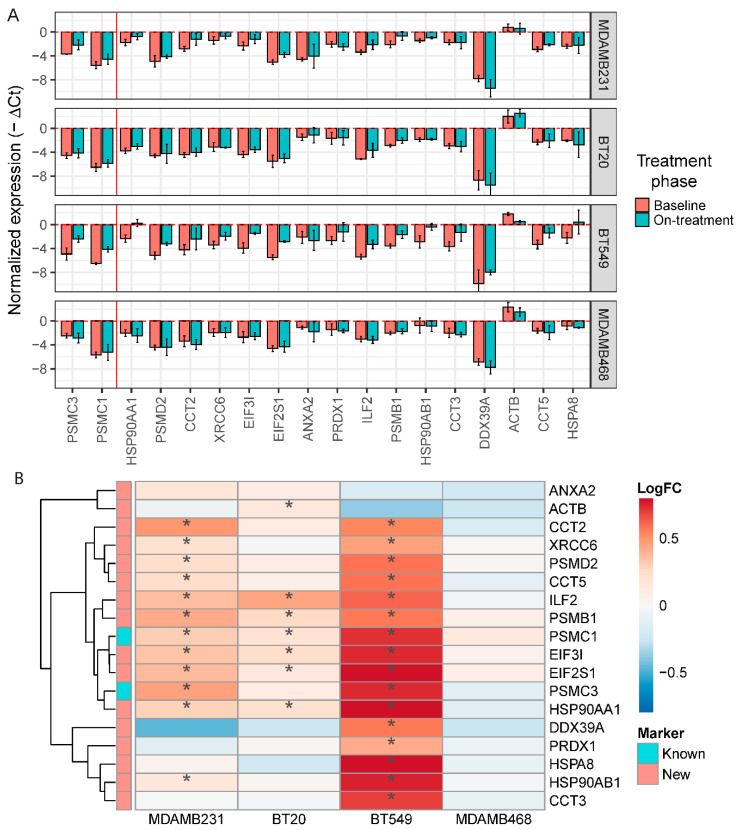
In vitro validation of new markers of resistance to navitoclax. (**A**) Normalized expression level (GAPDH as a reference) of 16 new and 2 known markers of resistance in 4 triple-negative breast cancer (TNBC) cell lines after navitoclax treatment. Error bars show mean expression with 95% confidence intervals. Colors represent phase of the treatment. Red vertical line separates known and new markers. Negative values on the plot represent the expression lower than GAPDH. (**B**) Log fold change of expression between baseline and navitoclax-treated cells. * represents significant (*p* < 0.05) increase of expression in navitoclax-treated cells.

To confirm the association between cell survival after navitoclax treatment and expression of the top overexpressed genes (ranked by FDR) in the navitoclax-resistant cell population, we treated four TNBC cell lines (MDA-MB-231, BT20, BT549, and MDA-MB-468) with 10 µM navitoclax for 72 h and assessed mRNA levels with qPCR. To maintain consistency between qPCR experiments and to avoid plate-to-plate variations during qPCR analysis, we chose one housekeeping gene for normalization (GAPDH), and two already known navitoclax-regulated genes, PSMC1 and PSMC3, and 16 novel markers for analysis. The average expression of these 18 resistance markers was significantly correlated with average expression of all genes overexpressed in resistant cells at the single-cell level (r = 0.78, *p* < 2.2 × 10^−16^) indicating that our 18 genes represent a broad spectrum of resistance markers. We next compared the expression levels of the 18 genes between baseline and post-navitoclax exposure in the four cell lines using qPCR. We observed a significant increase in PSMC1 and PSMC3 expression in three of the four cell lines and a nonsignificant increase in MDA-MB-468 cells (Figure 6A). MDA-MB-231 cells also showed significant upregulation of the 10 novel markers, confirming the single-cell data. BT20 cells showed upregulation of six novel markers compared to untreated (Figure 6B) and DMSO-treated control groups (Figure S5A). In BT549 cells, we observed significant upregulation of 14 out of the 16 novel markers. In contrast, none of the markers were significantly upregulated in navitoclax-treated MDA-MB-468 cells. In summary, out of the top 16 new markers analyzed, five were significantly enriched after treatment in three of the four cell lines, five in two cell lines, and five in one cell line; however, the expression trends were comparable between all cell lines (Figure 6B). The cell lines had different degrees of sensitivity to navitoclax. BT549 cells were the most sensitive, followed by MDA-MB-231 and BT-20, whereas the MDA-MB-468 cell line was the least sensitive (Figure S5B). Average expression of the 18 markers of navitoclax resistance in baseline samples was the lowest in the most sensitive cells and highest in most resistant cells (Figure S5C).

### 2.7. Validation of the 18-Gene Resistance Signature in the Cancer Cell Line Encyclopedia (CCLE) and Genomics of Drug Sensitivity in Cancer (GDSC) Databases

**Figure 7:**
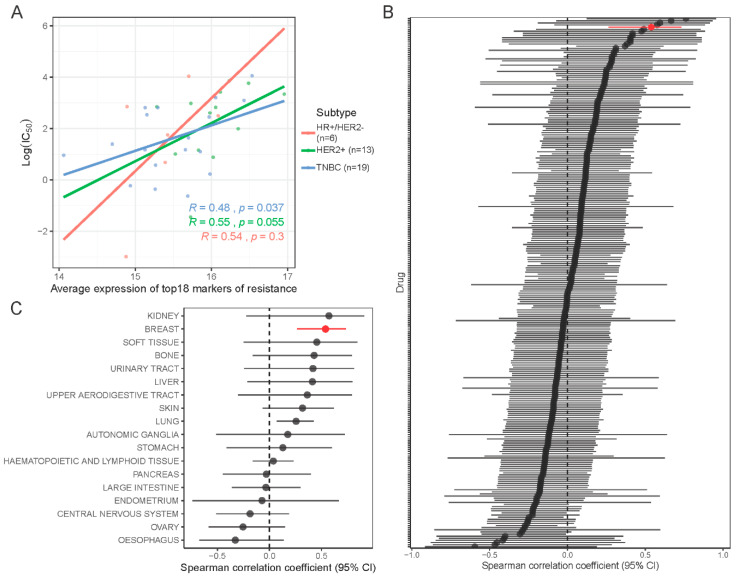
In silico validation of new markers of resistance in drug response database. (**A**) Association of signature of navitoclax resistance (18 genes) with log(IC_50_) drug response data from Cancer Cell Line Encyclopedia (CCLE) and Genomics of Drug Sensitivity in Cancer (GDSC) databases in 38 breast cancer cell lines grouped into 3 subtypes. Color lines show linear regression model fit. R is the Spearman correlation coefficient. (**B**) Forest plot showing correlation between signature of resistance and log(IC_50_) of each of the 251 drugs tested in breast cancer cell lines. Red line indicates navitoclax drug. (**C**) Forest plot showing correlation between signature of resistance and log(IC_50_) of navitoclax tested in 619 cell lines grouped in 18 tissues of origin.

Using gene expression and dose response data for 251 drugs in 619 cell lines from the CCLE and GDSC databases, we assessed the correlation between our proposed 18-gene resistance marker and sensitivity to navitoclax treatment. In 46 breast cancer cell lines, the average expression of the 18 resistance genes was highly correlated with the average expression of all markers of navitoclax resistance (Figure S6A). In breast cancer cells, the signature showed a modest but significant positive correlation with log(IC_50_) of navitoclax (r_S_ = 0.54, *p* < 0.01) and this correlation was higher than the correlation of 99% of individual genes with log(IC_50_) (Figure S6B). A similar association was observed in cell lines grouped into three breast cancer subtypes (Figure 7A), but there was greater variation. The 18-gene signature was not significantly correlated with response to other drugs, except tozasertib (Aurora kinase inhibitor) and NU7441 (DNA-dependent protein kinase inhibitor) (Figure 7B). In other cell types, we also observed a positive trend between higher expression of the 18-gene signature and lower sensitivity to navitoclax, but this reached statistical significance only in lung cancer (*N* = 107 cell lines) (Figure 7C and S6C).

### 2.8. Navitoclax Resistance Signature in the The Cancer Genome Atlas (TCGA) Human Samples

Using the gene expression data of 1096 breast cancer patients from TCGA, we calculated values of our 18-gene resistance signature for each patient and analyzed the signature within HR+/HER2− and TNBC subtypes. The distributions of signature scores were overlapping in the two subtypes (Figure S7) and covered a broad range of values. We hypothesize that cancers with high scores would be more resistant to navitoclax. On average TNBC had higher scores than HR+/HER2− cancers, suggesting that many TNBC patients might not be sensitive to navitoclax. In the absence of gene expression data of breast cancer patients treated with navitoclax, we cannot directly assess the association between our signature and response, but this could be tested as soon as such data become available.

## 3. Discussion

Cancer cells protect themselves from drug-induced cell death by upregulation of anti-apoptotic and pro-survival pathways, activation of DNA repair pathways, metabolic rewiring, epigenetic alterations, and autophagy. The adaptive mechanisms vary depending on the drug and the cancer type. We previously identified navitoclax as a potential therapy for TNBC and in this paper we examined the molecular effects of navitoclax treatment on a TNBC cell line using five different omics platforms to better understand the underlying adaptive resistance mechanisms.

After three days of navitoclax treatment, most differentially expressed genes were downregulated (*n* = 151), and only 26 genes showed elevated gene expression. We did not find any statistically significant changes in CNV, DNA methylation, and chromatin openness for 22 out of 26 upregulated genes. Increased expression of these 22 genes may be mediated through transcription factor-mediated mechanisms, or microRNA mediated post-transcriptional regulation that we have not studied. The differentially-expressed genes represent the transcriptional response in cells that survived the drug treatment for 72 h, including cells that are resistant to treatment and cells that are damaged but still surviving. The many more upregulated genes fell into broad biological pathways including “Myc targets”, “DNA repair”, “oxidative phosphorylation”, and “reactive oxygen species (ROS)”. This transcriptional response suggests some form of oxidative damage induced by navitoclax and a corresponding activation of repair and free radical quenching mechanisms [23,24,25]. ROS is produced by oxidative phosphorylation in mitochondria and therefore the observed increase in ROS pathways and oxidative phosphorylation could be direct effects of navitoclax. Excessive ROS also damages mitochondrial function by decreasing mitochondrial membrane potential and impairing ATP production [26]. Although navitoclax is not known to be genotoxic [27], apoptosis eventually leads to DNA fragmentation and the increased expression of DNA repair genes may represent an attempt to control early DNA fragmentation [28]. Intracellular ROS can also induce DNA damage, including double-strand breaks, that will active DNA repair. Upregulation of Myc in response to navitoclax is a novel observation. Myc regulates the expression of many genes involved in DNA synthesis and cell proliferation, both processes seen in surviving cells post exposure.

When the treated cells were allowed to grow and recover in drug-free media, we observed increased glycolysis with a concomitant decrease in oxidative phosphorylation. Elevated glycolysis is commonly seen in cancer cells after exposure to cytotoxic drugs and is considered to be an antecedent of increased proliferation [29]. Glycolysis provides precursors for biosynthesis and for antioxidant production through the pentose phosphate (PPP) and serine synthesis (SSP) pathways [30,31]. PPP produces ribose-5-phosphate for nucleotide synthesis, and regenerates NADPH to maintain redox metabolism [32]. Serine is the primary carbon donor to the tetrahydrofolate (THF) cycle, which is required for both purine and pyrimidine nucleotide biosynthesis during cancer cell proliferation [33]. Serine can also contribute to NADPH production via the folate cycle, which serves to maintain redox homeostasis and support anabolic reactions [34]. The SSP also produces reduced glutathione (GSH), which is a primary antioxidant in the cells [35]. Indeed, we observed decreases in both the ROS pathway and oxidative phosphorylation after recovery, which coincided with increased antioxidant production. HIF-1 alpha, and Myc are two important oncogenes and central regulators of metabolic rewiring in cancer cells [36,37]. Alterations in gene expression of these two pathways were observed in on-treatment (T2) and post-treatment (T3) groups, indicating a role in coordinating a shift from oxidative phosphorylation to glycolysis. Myc also downregulates BCL2 expression, which we observed after navitoclax exposure. Transcription factors associated with EMT that were upregulated in the recovery phase (T3) also induce changes in energy metabolism, cell motility, and proliferation [38].

Remarkably, most cells after drug-free recovery returned to their original pretreatment transcriptional and epigenetic states. However, we did find lasting changes in DNA methylation and copy number levels that could have been caused by eliminating sensitive cell clones with treatment, leading to slightly different genome structure and methylome compositions after 10 days of recovery from treatment. On the single-cell level we did not identify any subpopulations of cells that could represent new treatment resistant clones. The changes in gene expression are only partially explained by the differences in the methylation levels of promoter regions or chromatin openness in the various regions of genes. However, the overall difference in methylation level was negatively correlated with change in expression, whereas the difference in chromatin accessibility was positively correlated, as expected.

In this study, BCL2 expression was significantly reduced in surviving cells at T2 and T3 compared to baseline. This is consistent with recent observations suggesting that high BCL2 expression is associated with greater sensitivity to BCL2 inhibitors in preclinical, in vitro and in vivo models of lymphomas [39]. We confirmed upregulation of Mcl-1, a previously identified navitoclax resistance gene, in the drug-resistant cells detected by both single-cell analysis and also in the bulk RNAseq in on-treatment T2 samples (FDR = 0.038). Seven well-known anti-apoptotic genes, BIRC3, CDKN1A, GPX4, GSN, IER3, JUN, and TIMP1, were all significantly overexpressed in on-treatment (T2) samples compared to baseline, consistent with their pro-survival function. None of these expression changes were driven by de-methylation, chromatin opening, or gene amplification.

Resistant cells also showed higher expression of stemness markers, on average, than treatment sensitive cells. Cancer stem cells typically rely on oxidative phosphorylation over glycolysis for energy production. Overlap in pathway gene membership could in part explain the increased stemness of the resistant cells. Stemness is also increasingly viewed as a cell state, rather than a particular cell type, and cells expressing stem cell features have repeatedly been linked to drug resistance [40].

In this study, we identified several previously unknown markers of resistance to navitoclax treatment using single cell sequencing data (Table S3). We selected the top 18 genes from this list to construct a navitoclax resistance signature. We validated a negative association between this 18-gene signature and navitoclax sensitivity in three additional cell lines (other than the MDA-MB-231 cells used for discovery) including two sensitive and one resistant cell line in vitro. The signature was specific to navitoclax and correlated positively with drug response in a large panel of breast and lung cancer cell lines in the CCLE and GDSC databases.

Almost all genes involved in the 18-gene navitoclax resistance signature are involved in some form of cellular stress response, including protein folding and protein synthesis, DNA repair, and antioxidant functions. The two known markers, PSMC1 and PSMC3, belong to the 19s proteasome family. Suppression of these genes was shown to increase vulnerability to navitoclax [41]. In addition, we found that PSMD2 and PSMB1, members of the 26S and 20S proteasome families, which are part of the same protein degradation mechanism, are also predictive of resistance. These genes may coordinate the breakdown of damaged proteins and recycle amino acids for new protein synthesis [42,43]. Indeed EIF3I, EIF2S1, DDX39A, and ILF2 enable mRNA transcription, activate the translational machinery, and promote protein synthesis. PRDX1 is a key antioxidant enzyme, and HSPA8, HSP90AA1, HSP90AB1, and HSPA8 are heat shock proteins that refold damaged proteins after various insults. CCT2, CCT3, and CCT5 are also chaperon molecules involved in protecting newly-formed proteins from misfolding and aggregation under cellular stress. Another member of the 18-gene signature is XRCC6, which codes for Ku70 protein, which binds to DNA double-strand break ends and is required for DNA repair via the non-homologous end-joining pathway [44]. We also assessed the distribution of the score in breast cancers in the TCGA and found a broad range of values. We hypothesize that cancers with high scores would be more resistant to navitoclax, which can be tested in future clinical trials.

## 4. Materials and Methods

### 4.1. Cell Culture and Treatment

Triple-negative breast cancer (TNBC) cell lines BT20, BT549, MDA-MB-231, and MDA-MB-468 were purchased from the American Type Culture Collection (Manassas, VA, USA) and maintained in RPMI 1640 media and MEM (BT20 media) supplemented with 10% fetal bovine serum (FBS) and 1% penicillin-streptomycin. The cell lines were authenticated by the American Type Culture Collection (ATCC) regarding their origin and were not used beyond 6 passages. Navitoclax was obtained from Selleckchem, TX, USA (catalog no. S1001).

MDA-MB-231 cells were grown for two parallel sets of identical experiments (i.e., biological replicates). The entire experiment took 14 days, including 1 day to allow cells to grow and attach before starting navitoclax treatment. One million cells were plated in multiple 20-cm dishes corresponding to various time points (T1a—4 plates; T2a—20 plates; T3a—2 plates; T1b—4 plates; T2b—20 plates; T3b—2 plates). The number of plates differed per samples because samples T2a and T2b, after 3 days of drug treatment, had very few cells left in each plate, so we initially needed more plates to grow cells. For each time point and replicate, cells from multiple plates were pooled together and subsequently divided into different aliquots for molecular analyses (scRNAseq), bulk RNAseq, DNA methylation, CNV, and ATACseq. To minimize batch effects in library preparation, all samples were collected and processed on the same day by staggering the start date of experiments.

### 4.2. qPCR Experiments

Total RNA was isolated from untreated, DMSO-treated and navitoclax-treated cells using a RNeasy Plus kit (Qiagen, Hilden, Germany) according to the manufacturer’s instructions. Complementary DNA was synthesized using iScript cDNA synthesis kit (Bio-Rad, Hercules, CA, USA) according to manufacturer’s instructions. One microgram total RNA was used for synthesizing cDNA in 20-microliter reactions. qPCR analysis was done in a CFX96 Touch Real-Time System (Bio-Rad) using an iQ SYBR Green Supermix (Bio-Rad) with 50 ng of cDNA per reaction in triplicate. The qPCR primers used to analyze signature and housekeeping transcripts are listed in Table S4.

The relative expression of the target genes was quantified using Δ*CT* method with GAPDH as a reference gene. Negative value of ΔCT is proportional to target gene expression. Log fold change of expression was calculated using the 2^−ΔΔCT^ method by comparing expression in navitoclax-treated cells to baseline.

### 4.3. Cancer Cell Line Drug Response Database

Drug response data for 266 drugs tested in 1065 cell lines were downloaded from the Genomics of Drug Sensitivity in Cancer database (GDSC), release 7.0 [45]. The measure of drug response evaluated in the study was log(IC_50_), the drug dose (in log scale) required to reduce cell viability by half. Eight of the 26 tissues represented by less than 10 cell lines were removed from the analysis.

### 4.4. Gene Expression Data of Human Breast Cancer Samples

Summarized TCGA (The Cancer Genome Atlas) [46] RNA-seq expression data for 1096 breast cancer patients were obtained from the Pan-Cancer Atlas website (https://gdc.cancer.gov/about-data/publications/pancanatlas). Expression data were normalized using upper-quantile normalization and log_2_ transformed (log_2_(x+1)). The samples were assigned to 3 mutually exclusive subtypes including: (i) estrogen (ER) or progesterone receptor (PR)-positive and human epidermal growth factor receptor-2 (HER2)-negative (HR+/HER2− subtype); (ii) human epidermal growth factor receptor-2-positive with any ER or PR (HER2+ subtype); (iii) triple negative (TNBC subtype) based on immunohistochemistry (IHC) that was available. When IHC results were not available or were incomplete, we assigned the subtype based on mRNA expression levels of the estrogen receptor (ESR1) and HER2. The expression levels of these molecules show clear bimodal distributions that allowed fitting a two-component Gaussian mixture model to the data, including all breast cancer samples [47]. For each of the two genes, we defined the mRNA threshold to assign a positive or negative status as the intersection point between the two Gaussian functions, and samples with gene expression above this threshold were considered positive.

### 4.5. Genome and Trascriptome Annotation

To provide consistency across the different omics analyses, the same transcriptome annotation files were used across experiments, ENSEMBL release 84, and all sequencing reads were aligned to human reference genome hg38. Both annotation files were retrieved from Cell Ranger 2.1.1 software [48], and can be obtained from https://support.10xgenomics.com (accessed on 13 February 2020).

### 4.6. Single-Cell RNA Sequencing

Cells were trypsinized and single cell suspensions of 1000 cells/µL with viability ≥90% were processed using a Chromium Controller (10× Genomics, Pleasanton, CA, USA). A Chromium Single Cell 3′ Library and Gel Bead Kit V2 (PN-120237), Chromium Single Cell Chip A Kit (PN-120236) and Chromium i7 Multiplex Kit (PN-120262) were used to prepare single cell libraries. In total, 1500 cells per sample were sequenced in one lane of a HiSeq 4000 (Illumina, San Diego, CA, USA) flow cell at the Yale Center for Genomic Analysis (YCGA) to generate 300 M reads per 1500 cells. Raw RNA sequencing reads were processed with Cell Ranger 2.1.1 [48] to generate a gene-cell count matrix, which was analyzed with Seurat 2.3 (https://satijalab.org/seurat/). For analysis, we retained only cells expressing >1000 transcripts and <10% mitochondria transcripts, and only genes with non-zero expression in >10 cells. These thresholds were established with the help of GaMRed software [47].

For further analysis, we log-transformed each entry of the matrix by computing log_(CPM/100+1)_ values (CPM = counts per million). To visualize cell subpopulations, we used principal component analysis (PCA) followed by *t*-distributed stochastic neighbor embedding (t-SNE) [49] applied to the normalized data. Generalized Jensen–Shannon divergence, calculated with the gJSD function from the philentropy R package [50], was used to calculate the similarity of mRNA expression profiles between individual cells within each time point.

Cell clustering was done using partition-based graph abstraction (PAGA) method that provides an interpretable graph-like map (PAGA plot) of the arising data manifold, based on estimating connectivity of manifold partitions [51]. Prior to analysis, the data dimensionality was reduced to the first 20 principal components. A symmetrized graph was constructed with Euclidean distance measure and information from 7 nearest neighbors. The Louvain algorithm was used for reduced data clustering, with the resolution parameter set to 0.8.

Cell cycle analysis was performed in Seurat as follows. First, a score was calculated for each cell based on the expression of genes periodically expressed in human cell cycle states [52]. Second, a discrete classification of cell cycle stage was assigned to each cell by comparing scores calculated for five cell cycle states (G1/S, S, G2, G2/M, M/G1). Cells expressing neither were classified into the G0 group.

To assess the association between previously published navitoclax response genes and cell survival, two gene sets, “navitoclax targets” (*n* = 81) and “navitoclax resistance” (*n* = 61), were manually collated based on a literature search for studies of inhibitors of anti-apoptotic proteins (BCL2 family: Bcl-2, Bcl-XL, and Bcl-w) in in vitro, in vivo, and clinical settings (Table S2). Navitoclax-target genes were defined as direct and indirect targets of BCL2 family proteins, and cells that express high levels of these genes were considered navitoclax-sensitive. Navitoclax resistance genes were those reported to be overexpressed in cells not responding to treatment with BCL2 family inhibitors. Cells overexpressing these genes were considered navitoclax-resistant. “Target” and “resistance” gene scores were calculated for each cell using the same cell cycle state scoring algorithm in Seurat, which allowed us to assign cells into 3 categories; (i) resistant cells, if the navitoclax resistance score was positive and greater than the navitoclax target score; (ii) sensitive cells, if the navitoclax target score was positive and greater than the navitoclax resistance score; or (iii) other, if both scores were negative. To identify novel navitoclax resistance genes, the MAST R package [53] was used to find differentially-expressed genes between “resistant” versus “sensitive” or “other” cells (FDR < 0.01 and Hurdle model coefficient significant at 0.05 level).

### 4.7. Bulk RNA Sequencing

Total RNA was extracted from approximately 1 million cells using an RNeasy Plus Mini kit. RNA-seq libraries were prepared from 1 µg of purified total RNA suspended in nuclease-free 50 µL H_2_O using PolyA selection with oligo-dT beads, followed by random priming using the Illumina TruSeq Stranded Total RNA kit. Six samples were multiplexed in one lane of the HiSeq 4000 platform, generating 50 M 75 bp paired-end reads per sample. Raw RNA sequencing reads were assessed for quality using FastqQC v0.11.5 [54] and adapter sequences were removed using Trimmomatic v0.36 [55]. Remaining reads were aligned using STAR v2.5.3a [56] in two-pass mode and default parameters. Gene expression was quantified using RSEM v1.3.0 [57] with the option of a strand-specific RNA library. Only protein coding genes expressed in at least 1 sample were selected for further statistical analysis (16,334 genes). To find the genes that were differentially expressed (FDR < 0.05 and |log2 fold change| > 1) between the 3 time points, we used the DESEq2 R package [58]. Gene set enrichment in MsigDB [59] hallmark pathways and Nanostring hallmarks of cancer pathways (https://www.nanostring.com) were tested using the gene set enrichment analysis (GSEA) method implemented in the fgsea R package [60], using DESEq2 statistics as the gene ranking metric [61].

### 4.8. ATAC Sequencing

The ATAC library was prepared as described in [62]. Briefly, 50,000 cells per sample were used to prepare crude nuclei, followed by transposition reaction in a final volume of 50 µL. Tagmented DNA fragments were purified using a Qiagen Mini-Elute PCR Purification Kit and Illumina compatible libraries were generated by PCR using high fidelity 2× NEB master mix (NEB), as per manufacturer’s instructions. Cycling was done on a Bio-Rad C1000 thermal cycler with the following parameters: 72 °C for 5 min, 98 °C for 30 s, then five cycles of 98 °C for 10 s, 63 °C for 30 s, and 72 °C for 1 min. To reduce size bias and stop PCR saturation, the number of required cycles was determined by searching for the saturation point on time vs. the cycle number curve from qPCR. Next, the products were size-selected for 200 to 700 bp and were purified using a gel extraction kit (Qiagen). Six samples were multiplexed on one lane of a HiSeq 4000 and sequenced using a 75-bp paired-end read length, generating 50 M reads per sample. Raw sequencing reads were quality trimmed, and adapter sequences were removed with trimGalore [63] and aligned using bowtie2 [64] with default parameters. The duplicate reads were removed using Picard tools [65] and reads mapped to multiple regions of the genome were removed using SAMTools v1.9 [66] with the q ≤ 2 option. Peaks were identified for each sample separately using MACS2 [67] with the following options: --nomodel --nolambda --pvalue 1 × 10^−5^. BEDTools [68] was used to annotate peaks with gene information from gene transfer format (GTF) files. Each gene region was extended by 1000 bp up- and down-stream. Peaks located within these extended gene regions were included in further analysis. Differential peaks (FDR < 0.05 and |log2 fold change| > 1) were identified using the DESeq2 R package [58].

### 4.9. DNA Methylation

DNA was extracted using a DNeasy Blood and Tissue kit (Qiagen) and libraries were prepared from 1 µg of DNA using SeqCap Epi CpGiant Probes (Roche, Basel, Switzerland). Six samples were multiplexed in one lane and sequenced using 75 bp paired-end sequencing on the HiSeq 4000 platform, generating 50 M reads per sample. Whole-genome bisulfite sequencing reads were assessed for quality using FastqQC v0.11.5 [54], adapter sequences were removed with Trimmomatic v0.36 [55], and the remaining reads were aligned using bitmapperBS [69] with default parameters. The duplicate reads were removed using SAMTools v1.9 [66]. Methylation profiles were extracted using MethylDackel software (https://github.com/dpryan79/MethylDackel) with the following parameters: – –OT 3,0,0,99 – –OB 0,99,3,0, giving methylation metrics per CpG site. Only CpG sites overlapping regions of the genome targeted by the SeqCap Epi CpGiant protocol were retained in further analysis. CpGs that were not methylated in any sample, or were fully methylated in all samples, or had coverage of less than 10× were filtered. The remaining variably methylated CpGs were annotated using the Genomation R package [70]. To find differentially methylated CpGs (FDR < 0.05 and |difference in methylation level| > 0.2), the DSS R package was used [71]. Methylation level of gene promoter regions was calculated as a coverage-weighted average of methylation for CpG sites within 1000 bp up- or down-stream from transcription start site.

### 4.10. Copy Number Variants

Sequencing libraries were prepared from 1.5 µg DNA using the KAPA Hyper library prep kit (Roche). Sequencing was done by multiplexing six samples in one lane of the HiSeq 4000 platform, generating 50 M 75-bp paired-end reads per sample with 5× average coverage. Reads were aligned using BWA v0.7.17 [72] with default parameters. SAMtools v1.9 [66] was used to convert the sequence alignment map (SAM) files to binary alignment map (BAM) files, and then to sort and index them. Copy number variants (CNVs) were detected using CNVnator v0.3.3 [73], with a bin size of 500 bp and the -unique parameter. Putative CNVs were filtered by two criteria—length >1 kb and q0 (zero mapping quality) <0.15. The final CNVs were mapped to protein-coding genes using BEDTools v2.27.1 [68]. Profiles of CNVs were visualized using a circos plot with the BioCircos R package [74].

### 4.11. Data Availability

All omics data generated in this study have been deposited in Sequence Read Archive (SRA) under the project accession number PRJNA657088.

## 5. Conclusions

We performed a comprehensive analysis of navitoclax-induced molecular changes in triple-negative breast cancer cells and identified both transient and more lasting effects. Overall, the results highlight a very complex and large-scale, mostly reversible cellular response to navitoclax, with considerable between-cell variabilities. We developed a novel 18-gene expression signature of resistance to navitoclax that can used as a tool to predict navitoclax sensitivity, which will require validation in clinical samples.

## Supplementary Materials

The following are available online at https://www.mdpi.com/2072-6694/12/9/2551/s1, Figure S1: Reproducibility of measurements across modalities; Figure S2: Association of changes in expression level with other modalities; Figure S3. Partition-based graph abstraction (PAGA) analysis on single-cell expression data; Figure S4: Gene set enrichment analysis of resistant cells vs. other; Figure S5: In vitro validation of 18-gene navitoclax signature; Figure S6: In silico validation of 18-gene navitoclax signature; Figure S7: Navitoclax resistance signature in human breast cancer samples; Table S1: Significant genes and CpG sites; Table S2: Biologically relevant gene sets; Table S3: Markers of navitoclax resistance; Table S4: PCR primer list.
