# Supplementary material for: Multi-Omics Investigation of Innate Navitoclax Resistance in Triple-Negative Breast Cancer Cells
Source: Cancers (Basel). 2020 Sep 8;12(9):2551. doi: 10.3390/cancers12092551 (PMC7563413; doi:10.3390/cancers12092551)
Supplement: Supplementary file 1 [file cancers-12-02551-s001.zip › Supplementary Figures.pdf]

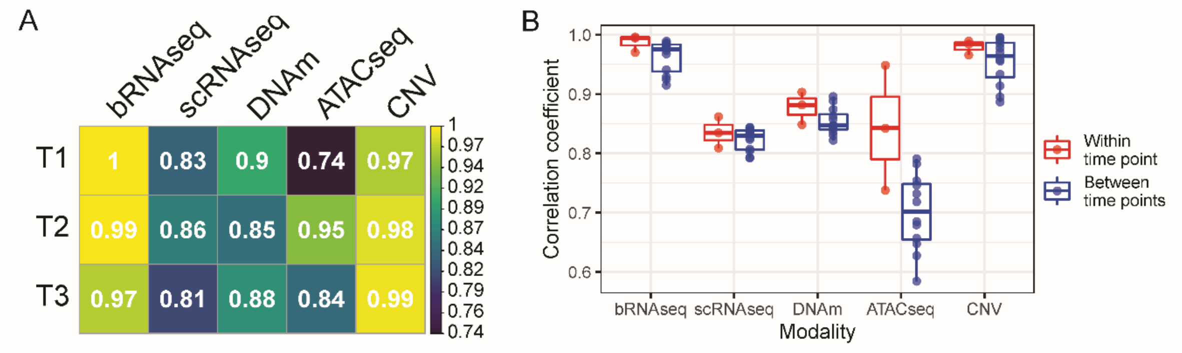

Figure S1. Reproducibility of measurements across modalities. (**A**) Pairwise Spearman correlation coefficients between biological replicates for each analysis type at each time point (T1: baseline, T2: on-treatment, T3: post-treatment). (**B**) Correlation coefficients between replicates at each time point (red dots) and between different time points (blue dots). For bulk RNAseq and CNV the high correlations caused overlap of dots.

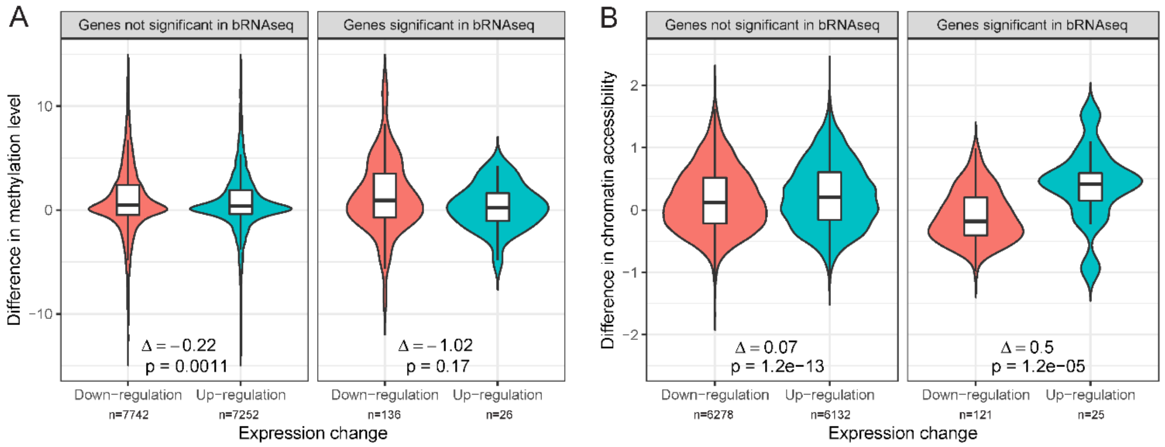

Figure S2. Association of changes in expression level with other modalities. Distribution of methylation level (**A**) and chromatin accessibility level (**B**) of gene down- and up-regulated on bulk expression level. On the left side there are not-significant and on the right side significant genes in comparison between baseline (T1) and on-treatment (T2) (FDR < 0.05).

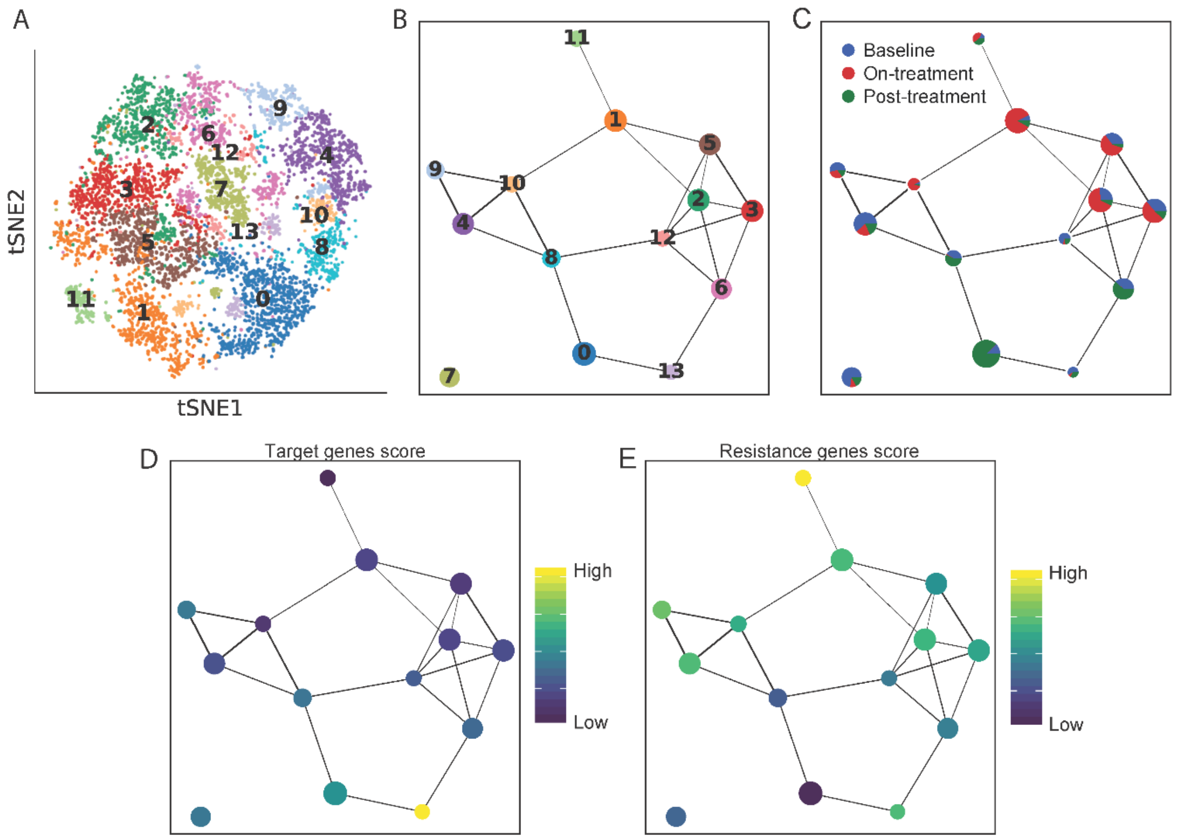

Figure S3. Partition-based graph abstraction (PAGA) analysis on single-cell expression data. (**A**) T-SNE plot of aggregated data colored by identified cluster. (**B**) PAGA graph colored by identified cluster. (**C**) PAGA graph with distribution of cells across 3 time points in each cluster. (**D**) PAGA graph colored by navitoclax target genes score. (**E**) PAGA graph colored by navitoclax resistance genes score.

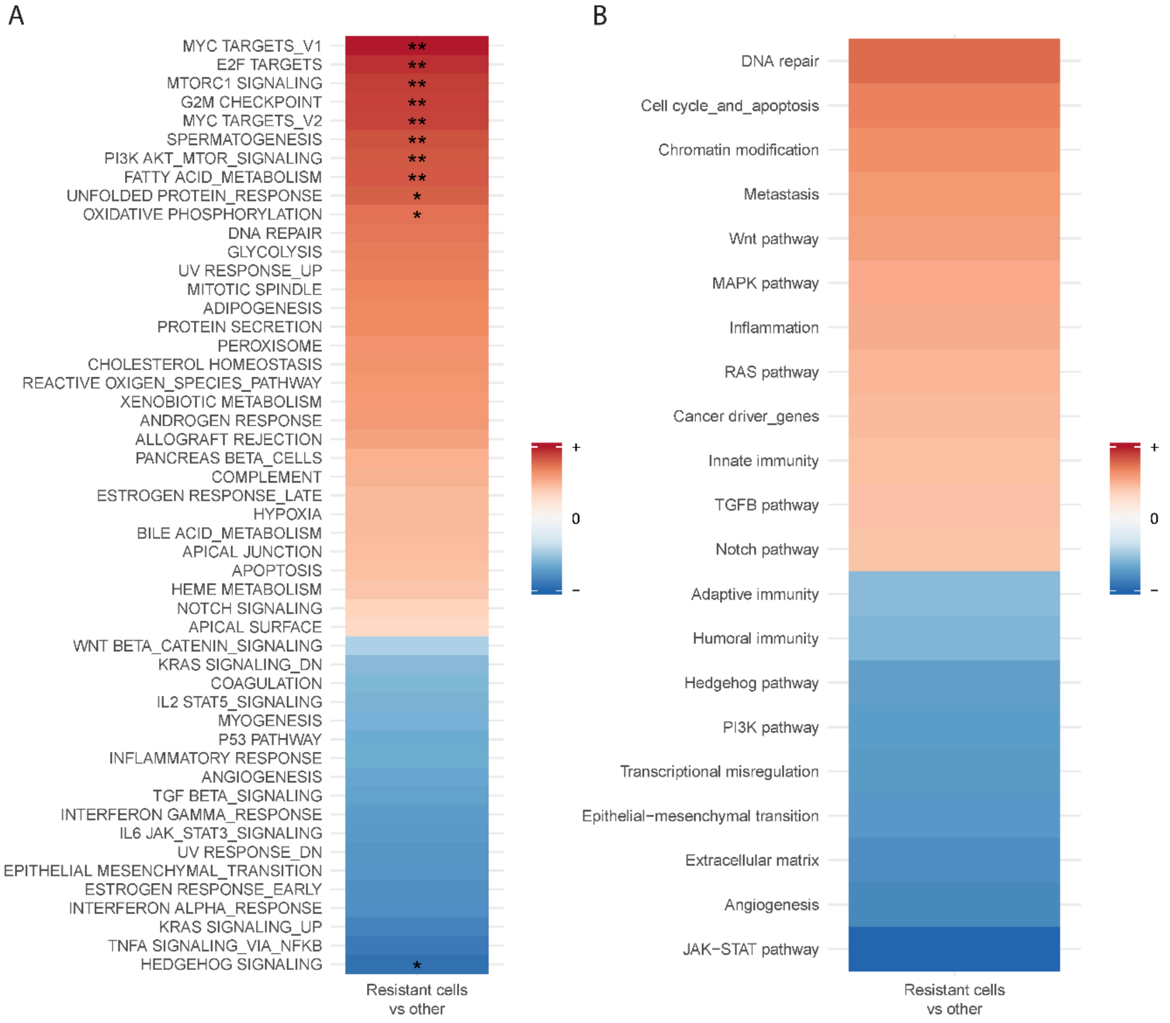

Figure S4. Gene set enrichment analysis of resistant cells vs other. Two gene sets collections were used: MSigDB hallmark genes (**A**) and Nanostring cancer hallmark pathways (**B**). Color indicates directionality (+ means higher in resistant cells) and stars indicate significant results (*adjusted p < 0.01, **adjusted p < 0.001).

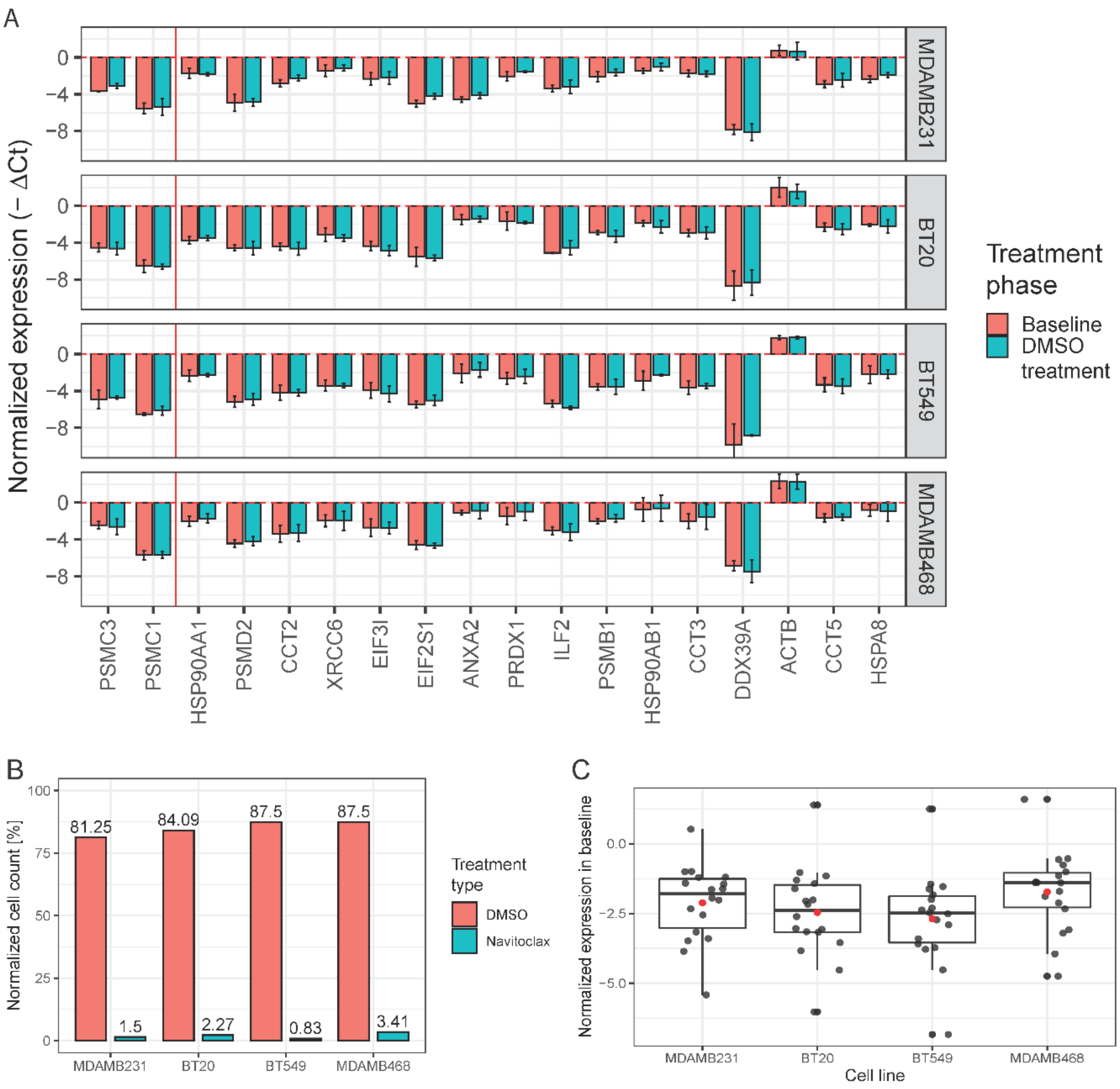

Figure S5. ***In vitro* validation of 18-gene navitoclax signature.** (**A**) Log expression level normalized to GAPDH (-dCt) of 16 new and 2 known markers of resistance in 4 TNBC cell lines after DMSO treatment. Error bars show mean expression with 95% confidence intervals. Colors represent phase of the treatment. Red vertical line separates known and new markers. (**B**) Percent of cells after 3 days of treatment normalized to untreated control. (**C**) Distribution of expression values of 18 markers of resistance in 4 cell lines. Red dots show the average value.

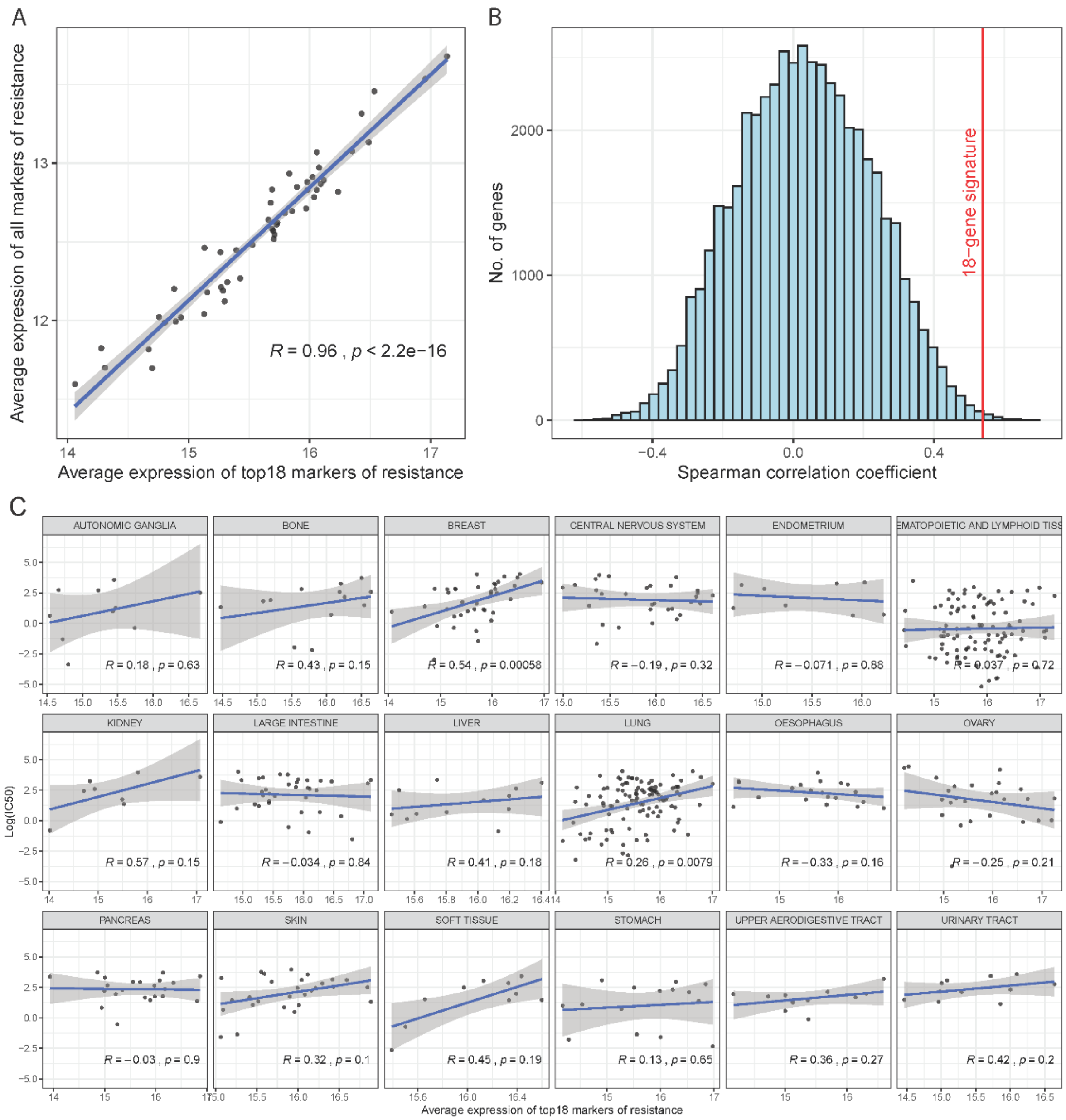

Figure S6. *In silico* validation of 18-gene navitoclax signature. (**A**) Association of signature of navitoclax resistance (18 genes) with the average expression of all markers of navitoclax resistance (2,364 genes) in 38 breast cancer cell lines. Blue line shows linear regression model fit with 95% confidence intervals. R is a Spearman correlation coefficient. (**B**) Distribution of Spearman correlation between individual gene expressions and log(IC_{50}) drug response data in breast cancer cell lines. (**C**) Association of signature of navitoclax resistance (18 genes) with log(IC_{50}) drug response data in 619 cell lines grouped into 18 tissues.

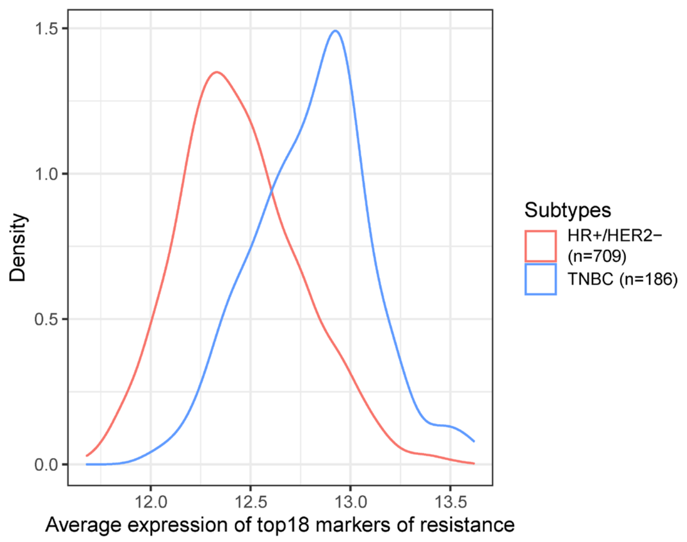

Figure S7. Navitoclax resistance signature in the human breast cancer samples. Distribution of signature of navitoclax resistance (18 genes) across breast cancer subtypes in TCGA data.
